# Editorial focus: entering into the non-coding RNA era

**DOI:** 10.1186/s11658-018-0111-3

**Published:** 2018-09-18

**Authors:** Rafal Bartoszewski, Aleksander F. Sikorski

**Affiliations:** 1 Department of Biology and Pharmaceutical Botany, Medical University of Gdansk, Gdansk, Poland; 2 Department of Cytobiochemistry, Faculty of Biotechnology, University of Wroclaw, Wroclaw, Poland

**Keywords:** ncRNAs, microRNAs, lncRNAs, Regulation of gene expression

## Abstract

Recent developments in high-throughput genotyping technologies have revealed the existence of several new classes of RNA that do not encode proteins but serve other cellular roles. To date, these non-coding RNAs (ncRNAs) have been shown to modulate both gene expression and genome remodeling, thus contributing to the control of both normal and disease-related cellular processes. The attraction of this research topic can be seen in the increasing number of submissions on ncRNAs to molecular biology journals, including *Cellular Molecular Biology Letters* (CMBL). As researchers attempt to deepen the understanding of the role of ncRNAs in cell biology, it is worth discussing the broader importance of this research.

The correlation between genotype and phenotype has become the Holy Grail of biomedical research in recent decades. High-throughput genotyping technologies such as deep sequencing have dramatically enhanced our understanding of the function and complex organization of the eukaryotic genome. They have also revealed the existence of several new classes of RNA that do not encode proteins, instead serving other cellular roles: the non-coding RNAs (ncRNAs) [1–4].

To date, ncRNAs have been assigned a variety of functions, including regulation of gene expression, both transcriptional (eRNAs) and post-transcriptional (microRNAs; miRNAs); modulation of RNA processing (ribozymes); translation (miRNAs); and protection from foreign nucleic acids (piRNA) [1–6]. Furthermore, ncRNAs can guide DNA synthesis or genome rearrangement. Looking at these functions from a high level, we can state that these ncRNAs modulate both gene expression and genome remodeling [1]. Although some classes of ncRNA simply utilize their RNA structure to serve their biological function (ribozymes and riboswitches), the majority require specific associations with proteins (snRNPs, snoRNPs, microRNAs, piRNA and long ncRNAs) to fulfill their biological activity [1, 6, 7].

The significant growth in number of reports on ncRNAs began in the early 2000s, when miRNAs, a class of short ncRNAs (22–25 nt), were accepted as post-transcriptional regulators of eukaryotic gene expression. The level of interest in these nucleic acids has certainly not abated. Recently, the editors of *Cellular Molecular Biology Letters* (*CMBL*) have witnessed a sharp increase in the number of submissions aiming to deepen the understanding of the role of ncRNAs in cell biology. In the light of this increased attention, we would like to briefly discuss that role here.

Without doubt, miRNAs constitute the most extensively studied class of ncRNAs. Discovered in the laboratory of Dr. Victor Ambros and Dr. Gary Ruvkun in 1993, the original studied organism was *Caenorhabditis elegans* [8–10]. MiRNAs are now known to be present in most eukaryotes, including humans [11–13]. It is estimated that they account for 1–5% of the human genome (from < 1 to 50,000 molecules per cell [14]) and regulate at least 30% of the protein-coding genes [15–17].

In mammalian cells, miRNAs recognize specific target mRNA sequences (with sites most frequently localized within the 3’-UTR) and initiate translational repression followed by decay of the mRNA [18–21]. However, since miRNAs rarely repress their target genes by more than 30%, recent reports suggest that their function is rather to modulate gene expression then to serve as strong post-transcriptional repressors [22]. Nevertheless, some miRNAs cause large-scale switch-like effects under stress or disease conditions [23–26]. Notably, mammalian miRNAs are also active in the nucleus, where they are proposed to mediate chromatin silencing at specific loci by base pairing to nascent transcripts [27–29].

Since mammalian miRNAs are not perfectly complementary to their target mRNA sequences, specific miRNAs can modulate transcriptional networks comprising numerous interdependent targets such as like transcription factors [30]. This means that a single miRNA generally modulates multiple mRNAs and that most mRNAs are modulated by multiple miRNAs [31, 32]. This makes it extremely challenging to decipher the precise molecular mechanisms underlying the biological function of any given miRNA.

Nevertheless, it is now evident that miRNAs play a crucial role in the regulation of gene expression, controlling diverse cellular and metabolic pathways [33–35]. Importantly, miRNAs contribute to the control of developmental differentiation [30, 36–41] and disease processes [42–50]. Compelling reports studies have demonstrated that the expression profiles of certain miRNAs, the so-called oncomiRs, are deregulated in human cancer, and that these ncRNAs have either oncogenic or tumor suppressor functions [51–57]. OncomiRs have been reported to affect cancer cell proliferation and signaling, prevent cell death, stimulate invasion and metastasis, and promote angiogenesis [52, 58–66]. Obviously, understanding the mechanism governing oncomiR cellular function could provide a basis for novel anticancer strategies.

Although oncology is a major source of reports on miRNAs, they are also recognized as important mediators of cellular responses to various stress stimuli, including the hypoxia that accompanies not only cancer but also cardiovascular disorders. This cellular insult modulates miRNA expression to restore oxygen homeostasis and survive hypoxic stress [67–70]. Recent reports have provided compelling evidence that miRNAs modulate hypoxic transcriptional networks, related angiogenesis and endothelial function [39, 53, 71–79]. Furthermore, many hypoxamiRs recently described in normal endothelium were previously reported as oncomiRs, suggesting that understanding hypoxia-related changes in miRNA profiles could be beneficial for both cardiovascular and cancer research [68, 69, 80–89].

Despite miRNAs being the most often examined class of ncRNAs, the biological role of long non-coding RNA (lncRNAs; > 200 nt) has become recently a popular but often controversial topic [90, 91]. To date, lncRNA have been implicated in a range of developmental processes, cellular stress responses and human diseases, including cancer [92–94]. They have been proposed to carry out a large number of biological functions and to modulate gene expression transcriptionally and post-transcriptionally through a variety of mechanisms that include: dueling polymerase activity [95–98], antisense RNA base pairing [99, 100], inhibiting histone acetyltransferase activity and repressing transcription [101, 102], recruiting transcriptional regulators [103–106], and chromatin remodeling [102]. Despite the ongoing increase in the number of reports describing lncRNA functions, our knowledge of the mechanisms by which they act remains limited. Novel and dedicated experimental methods to explore their mechanisms of action are needed [107].

There is no doubt that understanding the role and function of ncRNAs is crucial for the further development of cell biology and that it also may contribute novel therapeutic strategies for human pathologies [108]. However, achieving this challenging goal requires further experimental research. Unfortunately, a plethora of often contradictory reports of ncRNA roles exists, especially in the context of human diseases, with the majority utilizing computational and predictive methods, lacking any experimental verification and ignoring the necessity of the mechanistic approach.

Our goals with *CMBL* remain to reflect new and evolving advances in cellular and molecular biology, and to establish a record of accepting only reports of high-quality, innovative and state-of-the-art studies. Thus, we recommend that submitting authors consider two main criteria for reporting on ncRNA function: (i) functional experimental validation of the ncRNA related to its direct target or affected process; and (ii) mechanistic testing of the mechanism by which the ncRNA contributes to a disease or modulates cellular processes.

Notably, the means of engagement between miRNAs and their target mRNAs remains not fully understood. Even a single nucleotide change in either the miRNA or mRNA target sequence may have functional consequences [109–112]. That means that even sites strongly predicted as targets require functional validation.

Furthermore, while miRNA expression profiles often undergo dynamic changes due to epigenetic factors [113], the commonly used methods to confirm differential miRNA:mRNA binding, such as in vitro luciferase reporter assays and miRNA overexpression [114], often ignore the physiological miRNA levels in vivo. Importantly, changes in a particular gene’s mRNA level are not always definitively reflected in the protein levels [115]. Studies of ncRNA-affected targets should always be accompanied by monitoring of the protein levels.

Finally, ncRNAs can directly and simultaneously modulate multiple targets – and as with transcription factors, that means their signal can transfer onto a vast number of indirect effectors. A single miRNA is usually predicted to modulate hundreds of mRNAs and thus may have multiple effects on cellular metabolism. Therefore, predictions of the cellular function of ncRNAs require verification if the ncRNA effects are direct.

Recently, guidelines were proposed for the functional annotation of miRNAs using the Gene Ontology classification [116]. Similar recommendations will hopefully soon be available for other ncRNA classes. The nomenclature for ncRNAs has not kept up with developments in the field, which often contributes to disparities in ncRNA functional assignments. Moreover, the nomenclature requires redefining to provide clear discrimination between putative and bona fide ncRNAs and to include: isomiRNAs (variants of mature miRNAs), and organellar miRNA (like mitochondria specific mitomiRNAs) [117]. Although these limitations can be challenging to address experimentally, they should be carefully considered when proposing a biological function for ncRNAs or connecting them with a disease phenotype.

We trust that *CMBL* will further provide a environment to integrate novel, high-quality findings regarding the role of ncRNA in cell biology and human disease. We are inviting researchers to submit manuscripts on these topics fulfilling the above-mentioned criteria in the belief that our journal provides a professional forum to exchange knowledge and experience concerning ncRNA function in controlling cellular processes.

## Abbreviations

CMBL: Cellular Molecular Biology Letters
lncRNA: long non-coding RNA
miRNA: micro RNA
ncRNA: non-coding RNA
